# Investigating the causal relationship between employment and informal caregiving of the elderly

**DOI:** 10.1186/s13104-018-3684-z

**Published:** 2018-08-10

**Authors:** Edel Walsh, Aileen Murphy

**Affiliations:** Department of Economics, Cork University Business School, University College Cork, Cork, Ireland

**Keywords:** Informal care, Employment, Propensity score matching

## Abstract

**Objective:**

Examining the causal relationship between employment and informal caring to date has been impeded in countries like Ireland where there is a lack of suitable panel data and/or variables for instrument construction. This paper employs propensity score matching to control for non-random selection into treatment and control groups which controls for differences in employment outcomes between carers and non-carers in Ireland using data from Quarterly National Household Survey 2009 Quarter 3. Earlier papers focus on using regression techniques which may lead to biased estimates.

**Results:**

Results suggest that differences exist between carers and non-carers with respect to their employment status in Ireland. Overall the results suggest that the effects are more significant for those providing greater hours of informal care per week than those providing fewer hours of care per week. The effects estimated in this paper are likely to be more precise as failing to account for potential biases in the relationship are likely to underestimate the true effect of caring on employment outcomes. We find that propensity score matching provides an alternative method of examining the relationship when suitable panel data and/or variables for instrument construction are not available.

**Electronic supplementary material:**

The online version of this article (10.1186/s13104-018-3684-z) contains supplementary material, which is available to authorized users.

## Introduction

As well as being preferred by older people themselves and favourable for the public purse, informal care has been demonstrated to be a substitute for formal long-term care [1–4]. However, there are concerns as to whether supply will meet anticipated demands [2]. Supply of carers is restricted by social and economic changes such as increased female labour force participation, delayed retirements and migration, as well as changing family structures including smaller families, decreased co-residency of elderly with their children and the decline in traditional marriages. Therefore, it is feared a global shortage may arise [5, 6]. Previous research demonstrated that co-residential informal care does compete with other activities such as employment and childcare [7]. This is unsurprising given that informal carers are more likely to be women [8], who are married and have lower levels of education [9].

The relationship between informal caring and carers’ employment or labour market outcomes is complex, and despite extensive research ambiguity remains [10]. While results are varied, the majority of research concludes that caregiving exerts modest negative pressure on the likelihood of formal employment and hours worked, suggesting a trade-off between caring and working [2, 8, 10–12]. Recent research has found varying effects by age [13], gender and occupation [14, 15]. Dealing with this causal relationship between formal employment and informal caregiving is a recurring challenge in the literature. A variety of methods, including instrumental variables [10, 16–19]; difference in difference approaches [20]; simultaneous equation models [21, 22]; reduced form equations [23]; discrete time survival models [24] have been utilised to estimate the relationship between informal care and employment. However, employing these methods are dependent on data availability to construct instruments and/or utilising panel data. So, in countries like Ireland, where there is a lack of suitable panel data and/or variables for instrument construction, examining the causal relationship between employment and informal caring is impeded.

Leigh [25] attests that if it were possible to demonstrate if all carers were relieved of their caring duties would they have the same employment rate as non-carers, then cross sectional studies could provide unbiased estimates of the casual impact of caring on paid employment. This paper investigates Leigh’s [25] proposal and employs propensity score matching to determine if differences in employment outcomes between carers and non-carers exist in Ireland, where it is expected that by 2041 over 22% of the population will be over 65 years of age.

## Main text

### Data

The data used for this study is obtained from the Quarterly National Household Survey 2009 (n = 21,542). The Carers Module was a special module included in Quarter 3 of this survey, the focus of which was on those providing care (the ‘carer’) rather than those receiving care (the ‘dependent’). The data specifically asks about caring informally (unpaid) for others; that care being provided to anyone who has a long-term physical or mental illness or disability or problems related to old age. Given that we are interested in elder care provision, we confine our analysis to those providing care to individuals aged 65 and over and exclude the remaining carers (n = 578).

Table 1 presents the proportion of observations falling within each category for the full sample (n = 20,964). All respondents were asked about their employment status and the categories indicated if an individual is in paid employment (50%), unemployment (8%), or economically inactive (40%). The latter group includes students (4.2%), homemakers (21.3%), sick (3.5%), other-unspecified (0.5%) and those that are retired (12.2%). Since providing care is unlikely to affect the employment outcomes of this cohort they are excluded from our analyses. 6% of the full sample were carers and of those 57% provided less than 15 h of care per week and 43% care for 15 h or more per week.

**Table 1:** Descriptive statistics (*Source* QNHS, Q3, CSO (2009))

Variables	Description of variables	N (%)
Caring variables		
Carer	1 if engaged in informal caring duties, 0 if not caring	1394 (6)
< 15 h/week	1 if caring for less than 15 h per week, 0 if not caring	798 (4)
> 15 h/week	1 if caring for more than 15 h per week, 0 if not caring	539 (3)
Care 15 h	1 if caring for more than 15 h per week, 0 if caring for less than 15 h per week	596 (43)
Employment status		
Employed	1 if employed, 0 otherwise	10,447 (50)
Unemployed	1 if unemployed, 0 otherwise	1741 (8)
Personal characteristics		
Female	1 if female, 0 if male	12,360 (58)
Under 25	1 if under 25 years old, 0 otherwise	3353 (16)
Age 25–44	1 if between 25 and 44 years old, 0 otherwise	8714 (42)
Age 45–64	1 if between 45 and 64 years old, 0 otherwise	6122 (29)
Over 65	1 if over 65 years old, 0 otherwise	2775 (13)
Education level		
Primary	1 if primary school level only completed, 0 otherwise	4332 (21)
Second level	1 if secondary school level completed, 0 otherwise	7509 (36)
Post sec	1 if post-secondary/third level education completed, 0 otherwise	9123 (43)
Marital status		
Never married	1 if single/never married, 0 otherwise	6663 (32)
Married	1 if married, 0 otherwise	11,314 (54)
Widowed	1 if widowed, 0 otherwise	1652 (8)
Divorced	1 if divorced, 0 otherwise	466 (2)
Separated	1 if separated, 0 otherwise	868 (4)
Region in Ireland		
Border	1 if living in the Border region, 0 otherwise	2263 (11)
Mideast	1 if living in the Mideast, 0 otherwise	2219 (11)
Midland	1 if living in the Midlands, 0 otherwise	1108 (5)
Midwest	1 if living in the Midwest, 0 otherwise	2079 (10)
Southeast	1 if living in the Southeast, 0 otherwise	2353 (11)
Southwest	1 if living in the Southwest, 0 otherwise	3842 (18)
West	1 if living in the West, 0 otherwise	2036 (10)
Dublin	1 if living in Dublin, 0 otherwise	5064 (24)

N = 20,964. Categorical variables have a minimum value of 0 and a maximum value of 1

### Methods

This paper examines how hours of informal caring affect labour force participation. Owing to the fact that the relationship between these two variables is likely to be endogenous, regression techniques, such as ordinary least squares (OLS), may not be suitable in addressing the relationship. As an alternative we employ matching estimators to investigate if any differences exist with respect to labour force participation between groups of carers and non-carers, taking into account the hours of caring undertaken. Thus, we examine if all carers were relieved of their caring duties would they have the same probability of being employed as their non-carer counterparts. Propensity score matching techniques are based on the idea of comparing the outcomes of people that are as similar as possible with the sole exception of their treatment status. In this case ‘treatment status’ is whether the individual is a carer or not and the ‘outcome’ is employment status. More specifically, the analysis looks at caring (distinguishing between caring for more or less than 15 h per week) and employment status (employed or unemployed) to fully estimate the relationship between caring and labour force participation.

The type of propensity score matching used in this analysis is nearest neighbor matching. Here the individual from the comparison group (a non-carer) is chosen as a matching partner for a treated individual (a carer providing more than/less than 15 h of care per week) that is closest in terms of propensity score. The propensity score is defined as the conditional probability of receiving the treatment given the independent variables. Propensity score methods normally comprise four major steps [26]. Firstly, a propensity score is estimated for each unit using a probit regression of treatment conditions on covariates. Secondly, each unit in the treatment group is matched with one or more units in the comparison group based on the closest distance between their propensity scores. At this stage the balancing property must be satisfied. Thirdly, estimate the matching quality and finally, estimate the intended outcome analysis on the matched data or on the original data with propensity score adjustment or weighting [26]. The nearest-neighbor matching estimator calculates the average treatment effect (ATT) and the average treatment effect on the treated (ATET).

The matching procedure requires that the outcome variable (employment status), must be independent of treatment (caring) conditional on the propensity score [27]. Therefore, using matching requires choosing a set of independent variables (Xs) that satisfy this condition. Only variables that influence simultaneously the participation decision and the outcome variable should be included [28]. If P(X) = 0 or P(X) = 1 for some values of X matching cannot be used on those X values to estimate a treatment effect because individuals with such characteristics either always or never receive a treatment. According to Heckman et al. [29] some randomness is needed that guarantees that persons with identical characteristics can be observed in both states. To satisfy this condition several control variables are used in the analysis (specifically; gender, age, educational achievement, marital status and region) which may affect both the probability of providing informal care and employment status, respectively.

### Results

The probit estimates of care hours, which are used to calculate the propensity scores, are presented in Additional file 1: Table S1. These results suggest that participation in the treatment (being a carer) is significantly associated with a number of personal and socio-economic characteristics. The balancing property is satisfied for all matching estimates. The matching results are presented in Table 2. The results vary by hours of informal care provided and by outcome (labour force participation).

**Table 2:** Propensity score nearest neighbour matching

Outcome	More than 15 h care/week		Less than 15 h care/week	
	Number of observations treated (controls)	ATT (Std. Err^a^)	Number of observations treated (controls)	ATT (Std. Err*)
Employed	539 (12,309)	− 0.050 (0.020)***	798 (16125)	0.022 (0.017)
Unemployed	539 (12,309)	− 0.014 (0.010)*	798 (16125)	0.002 (0.009)

Average treatment effects on the treated (ATT). The numbers of treated and controls refer to actual nearest neighbour matches. Treatment (T) is carer hours > 15 h p/w (1, 0) or < 15 h p/w, Outcome (Y) is employed (1, 0) unemployed (1, 0). Independent variables are gender, age, education, marital status, region

*** Significant at the 1% level, ** Significant at the 5% level, * Significant at the 10% level

^a^Bootstrapped standard errors

The matching results show significant differences in employment outcomes between carers who care for more than 15 h per week and non-carers. The treatment effects are statistically significant across the employment outcomes suggesting that caring for more than 15 h per week has a significant effect on an individual’s probability of being in employment or unemployed (actively seeking work). The ATT coefficient between caring and employment is negative and significant, suggesting that caring for more than 15 h has a negative impact on an individual’s probability of being in employment (compared with not being a carer). Similarly, compared with being a non-carer, the ATT on unemployment is negative and significant (10% level) suggesting a negative relationship between caring for more than 15 h and those in unemployment (i.e. individuals seeking employment).

For those caring less than 15 h per week the balancing property is again satisfied however, results are insignificant. Overall, the results suggest that the effects are more significant for those providing greater hours of informal care per week than those providing fewer hours of care per week. The effects estimated in this paper are likely to be more precise given the potential biases in the relationship that are likely to underestimate the true effect of caring on employment outcomes.

The matching procedure is then carried out to control for differences in carers that care for more than 15 h per week and those caring for less than 15 h per week. More specifically, we are now comparing employment outcomes for groups of carers rather than comparing with non-carers. The results are presented in Table 3.

**Table 3:** Propensity score nearest neighbour matching

Outcome	Hours of caring per week (more than 15 h)	
	Number of observations treated (controls)	ATT (Std. Err^a^)
Employed	596 (692)	− 0.111 (0.029)***
Unemployed	596 (692)	0.007 (0.014)

Average treatment effects on the treated (ATT). The numbers of treated and controls refer to actual nearest neighbour matches. Treatment (T) is carer hours > 15 h p/w (1) > 15 h p/w (0). Outcome (Y) is employed (1, 0), unemployed (1, 0). Independent variables are gender, age, education, marital status, region (the variables ‘postsec’ and ‘southwest’ are dropped to satisfy the balancing property)

*** Significant at the 1% level, ** Significant at the 5% level, * Significant at the 10% level

^a^Bootstrapped standard errors

The ATT coefficient between hours of caring (> 15 h per week) and employment is negative and significant, suggesting that caring for more than 15 h has a negative impact on an individual’s probability of being in employment (compared with those caring for less than 15 h per week). We do not find a significant effect between caring and unemployment for different groups of carers.

### Discussion

A link between employment status and hours of informal care is established [10, 11, 16, 17, 21–23]. The usual techniques for handling the relationship between informal care and employment require panel data or construction of instruments. When these are not available investigating this link is difficult. This study illustrates when only cross sectional data is available, a matching estimator can be utilised to address the link between employment decisions and informal care provision across the two groups (carers and non-carers).

In this study the results differ across outcome (employed/unemployed) and treatment (care hours). The matching estimators are statistically significant for the employed and the unemployed which suggests that differences exist between carers of the elderly (> 15 h per week) and non-carers with respect to their employment outcomes. Further analyses confirm that differences also exist between groups of carers. Specifically between carers who care for more than 15 h per week and those that care for less than 15 h per week.

### Conclusion

As the demand for elder care increases, informal carers appear to be a “cheap” solution from the perspective of the health and social care budgets to elder care [1]. However relying on informal care requires informal carers, the supply of which are limited and unsustainable [2]. Thus, in planning for the future, the causal relationship between employment and informal caregiving needs to be examined. Such an investigation on a per country basis is important as the relationship between caring and employment is not consistent across countries owing to background and local structures [30]. To date such analyses has required panel data and/or variables for instrument construction. While data collection systems and efforts are improving, such data is not always available. This paper investigates Leigh’s [25] proposal that if it were possible to demonstrate if all carers were relieved of their caring duties would they have the same employment probability as non-carers, then cross sectional studies could provide unbiased estimates of casual impact of caring on paid employment. We find that propensity score matching provides an alternative method of examining the relationship when suitable panel data and/or variables for instrument construction are not available.

## Limitations

We acknowledge the data employed in this study is from 2009. However, the carers’ module has not been included in subsequent Quarterly National Household Budget Surveys; and the more recent Irish Longitudinal Study on Ageing (TILDA) data is confined to those over 50 years of age. Therefore the data represents the best available and is suitable for investigating the feasibility of using propensity score matching as alternative method of examining the relationship between caring and employment.

## Additional file

**Additional file 1.** Probit estimates of the probability of hours of informal care provided per week (used in the calculation of the propensity scores).

## Abbreviations

ATT: average treatment effect (ATT)
ATET: average treatment effect on the treated (ATET)
