# Supplementary material for: Investigating the causal relationship between employment and informal caregiving of the elderly
Source: BMC Res Notes. 2018 Aug 10;11:570. doi: 10.1186/s13104-018-3684-z (PMC6086034; doi:10.1186/s13104-018-3684-z)
Supplement: Supplementary file 1 — Additional file 1. Probit estimates of the probability of hours of informal care provided per week (used in the calculation of the propensity scores). [file 13104_2018_3684_MOESM1_ESM.docx]

**Additional file 1: Table S1.** Probit estimates of the probability of hours of informal care provided per week (used in the calculation of the propensity scores)

|  | **More than 15 hours of informal care per week** | | **Less than 15 hours of informal care per week** | |
| --- | --- | --- | --- | --- |
| **Variables** | **Coefficient** | **Std. Err.** | **Coefficient** | **Std. Err.** |
| **Personal characteristics** |  |  |  |  |
| Female | 0.305*** | 0.042 | 0.210*** | 0.035 |
| Under 25 years | -0.950*** | 0.121 | -0.426*** | 0.067 |
| 45-64 years | 0.192*** | 0.045 | 0.092** | 0.039 |
| Over 65 years | 0.247*** | 0.065 | -0.196*** | 0.068 |
| **Education Level** |  |  |  |  |
| Primary Level | -0.056 | 0.053 | -0.227*** | 0.049 |
| Post-Second Level | -0.188*** | 0.045 | ^ | ^ |
| **Marital Status** |  |  |  |  |
| Never Married | 0.077 | 0.047 | -0.145*** | 0.044 |
| Widowed | -0.776*** | 0.109 | -0.018 | 0.071 |
| Divorced | -0.099 | 0.126 | -0.035 | 0.103 |
| Separated | -0.058 | 0.090 | -0.145* | 0.084 |
| **Region in Ireland** |  |  |  |  |
| Border | -0.074 | 0.072 | -0.070 | 0.061 |
| Mideast | -0.261*** | 0.081 | -0.059 | 0.059 |
| Midland | -0.090 | 0.096 | 0.061 | 0.074 |
| Midwest | -0.032 | 0.072 | -0.087 | 0.063 |
| Southeast | -0.095 | 0.070 | 0.026 | 0.056 |
| Southwest | 0.050 | 0.056 | -0.134** | 0.052 |
| West | 0.053 | 0.069 | -0.057 | 0.063 |
| Constant | -2.015*** | 0.062 | -1.725*** | 0.046 |
| Observations | 20166 |  | 20425 |  |
| Pseudo R^2^ | 0.06 |  | 0.03 |  |

Notes: ***significant at the 1% level, ** significant at the 5% level, * significant at the 10% level. Base categories: male, 45-44 years, second level education, married, living in the Dublin region. Dependent variables: Carer by hours of care per week (1), Non-Carer (0). ^ post-secondary level education was omitted in order to satisfy the balancing property.
